# Is the Skin Absorption of Hydrocortisone Modified by the Variability in Dosing Topical Products?

**DOI:** 10.3390/pharmaceutics10010009

**Published:** 2018-01-12

**Authors:** Daniel A. Paterson, Jacqueline Hallier, Elizabeth Jenkins, Sarah F. Cordery, M. Begoña Delgado-Charro

**Affiliations:** Department of Pharmacy and Pharmacology, University of Bath, Bath BA2 7AY, UK; danielalbertpaterson@gmail.com (D.A.P.); Jacquihallier@hotmail.co.uk (J.H.); lizziejenkins42@hotmail.com (E.J.); S.Cordery@bath.ac.uk (S.F.C.)

**Keywords:** fingertip unit, dosing, variability, hydrocortisone, skin absorption, tape-stripping, topical corticosteroids, patient education

## Abstract

Fingertip units have been proposed as a tool to standardize topical therapy with semisolid formulations. However, no studies to date have characterized the variability in dosing by patients using this concept and whether this variability ultimately affects the topical absorption of drugs. This work aimed to answer these two questions. A first study determined the dose measured, the area of spread and the area-normalized dose for a 1% hydrocortisone cream and ointment applied by members of the public using this dosing approach before and after brief counselling. Then, in vivo tape-stripping and in vitro permeation studies investigated whether the variability in the area-normalized dose altered the skin absorption of hydrocortisone. Participants applied greater doses and spread them over larger areas after a short counselling intervention leading to smaller area-normalized doses. In vivo hydrocortisone uptake by the stratum corneum was significantly greater for the higher normalized dose and the differences were further supported by the in vitro permeation studies. However, these differences were relatively small and not proportional to the increase in normalized dose. This work shows that, following brief advice, patients and carers can apply consistent and sufficient doses of corticosteroids whilst minimizing risks and variability in hydrocortisone absorption.

## 1. Introduction

Applying topical dosage forms, particularly those with semisolid consistency, is not straightforward. From a patient point of view and in contrast with tablets and capsules, the dose administered with creams and ointments is not evident [1]. In fact, concerns regarding overdosing contribute to so-called “topical corticosteroid (TCS) anxiety” and poor adherence [2,3,4,5]. Low adherence has been associated with prescriptions with unclear instructions about “how much product should be used” and “the area of spread” [1]. Further, according to the Dermatology Working Group, current British National Formulary (BNF) guidance and patient information leaflets (PILs) (“apply TCS products sparingly and thinly”) convey a risk message, contributing to under-dosing and reduced adherence [2,6]. For example, a study found that on average, dermatologic patients applied only 35% of the expected individualized dosages [7]. The same Working Group suggested that provision of clear Fingertip Unit (FTU) instructions in TCS products and improved patient education in correct application of topical products could alleviate corticosteroid anxiety [1,2,3,5,7,8,9] and importantly, avoid under-dosing.

FTUs were proposed to facilitate doctor-to-patient communication about how to apply topical treatments [4,10,11]. The concept of FTU was based on the amount of formulation required to cover, thinly and evenly, specific body surface areas [4,10,11]. One FTU is the amount of formulation expressed from a tube with a 5 mm diameter nozzle, applied from the distal skin-crease to the tip of the index finger and corresponds to 0.49 g (for males; 0.43 g for females) of formulation that should be spread to cover 312 cm^2^ (for males; 257 cm^2^ for females) of skin or 2.14 (for males; 2.17 for females) flat hand areas [10,11]. A physician can, therefore, estimate the number of “flat hand” areas of skin involved and prescribe the number of FTUs required. However, little is known about how lay people understand FTUs, whether FTU dosing is efficiently communicated through short counselling (as possible in a busy clinical practice or community pharmacy), the variability associated with this dosing method and its potential impact on skin absorption.

It can be argued that inconsistency in dosing topicals results primarily in a variable area-normalized dose (i.e., different amounts of formulation applied per unit area of skin) or, in other words, layers of applied formulation with different thicknesses. The effect that application thickness of topicals has on skin absorption has rarely been addressed [12,13,14]. According to this prior work, as thickness increases, more material is placed on the skin per unit area so the active can be delivered over longer periods of time. Thin and thick films provide initially the same permeation rates but thin layers will deplete more rapidly leading to declining fluxes. Thus, the ratio (M_t_/M_∞_) or amount of active penetrating the stratum corneum (SC) at a given time (M_t_) relative to the total amount penetrating the SC at infinite times (M_∞_) approaches the value of one faster for thinner applications. To summarize, differences in the amount of drug penetrating the skin from different thicknesses are small in the beginning but become more apparent with time [14]. However, the magnitude of this effect over the range of formulation thicknesses applied by patients and its relevance with regard to TCS therapy has not been characterized.

This work addresses these questions. First, lay subjects were asked to apply one FTU of a hydrocortisone (HC) cream and ointment before and after a very short (<1 min) explanation of the concept. This study provided information about variability in dosing creams and ointments by lay subjects which was used to inform the subsequent steps. An in vivo study used tape-stripping methodology to measure the amount of hydrocortisone incorporated into the stratum corneum following thin and thick applications of the same formulations. Finally, the results of the latter were complemented by in vitro permeation studies involving a longer exposure time.

## 2. Materials and Methods

### 2.1. Materials

Hydrocortisone (≥98%), PBS (phosphate buffered saline, pH 7.4; Sigma, Leicestershire, UK), and high-performance liquid chromatography (HPLC) grade methanol were from Sigma, Leicestershire, UK. The 1% HC (hydrocortisone) ointment and the 1% HC cream were from Teva UK Ltd., Castleford, UK.

### 2.2. Inter-Subject Variability in Dosing Topical Products

**Figure 1:**
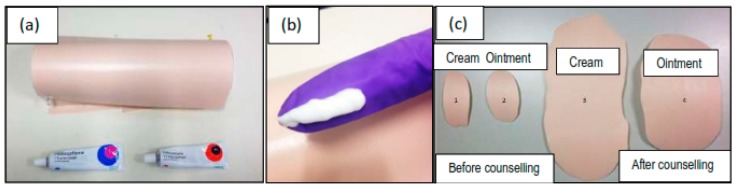
(**a**) model arm and formulations; (**b**) example of fingertip of formulation measured by one participant (**c**) representative set of areas used by one participant. From left to right: cream before counselling, ointment before counselling, cream after counselling, and ointment after counselling.

The study was approved by REACH (research ethics committee for the School for Health of the University of Bath, REACH; EP12/1316). Twenty-four healthy volunteers (Table 1) signed informed consent before participation. A questionnaire verified that the subjects’ specialism did not provide knowledge of FTUs and whether they had previous experience in applying TCS. First, the participants were asked to measure a fingertip unit of the cream without further information (group A) or being provided with the PIL (group B). The length of formulation expressed from the tube by the participants was measured. Next, the participants were asked to apply the formulation on a model arm and cover the area they considered appropriate (Figure 1). The process was repeated for the ointment. In a second step, the participants were briefly counselled by the researcher who simply described the British National Formulary (BNF) guidance (“*One FTU is measured by squeezing a line of cream (or ointment) from the tip of your index finger to the first crease. This is enough to cover an area of skin the size of your hand with the fingers together, twice. One FTU would be used to cover the front and back of one hand*”) [6] and asked the subjects to repeat the application process. Finally, the length of the participants’ fingertip was measured. To minimize HC exposure, the subjects wore gloves and a model arm (a cylinder wrapped in thin foam) (Figure 1) was used.

The areas of foam over which the products had been spread were wiped with dry tissue (Wypall, Kent, UK) to recover the formulation. The dose or amount of formulation applied was estimated as:

Dose (mg) = Weight wiped off model arm (mg) + Residual formulation on glove (mg)(1)

The section of foam was cut out, weighed and its area estimated through a calibration curve. The area-normalized dose (ND) was calculated as:

ND (mg/cm^2^) = Formulation applied (mg)/Area (cm^2^) (2)

**Figure 2:**
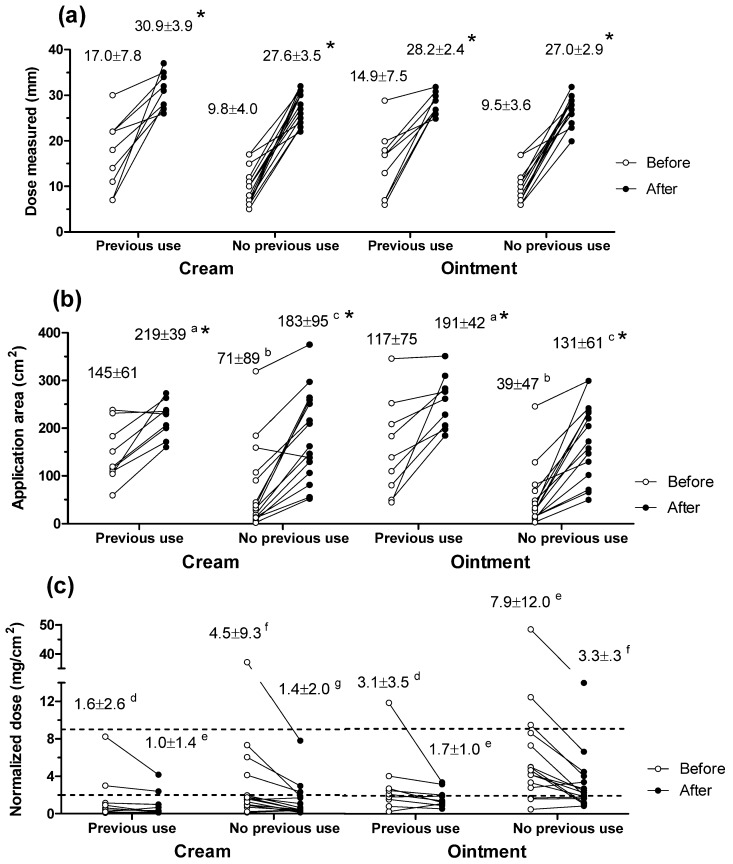
Mean (±SD) (**a**) dose measured; (**b**) area of spread and (**c**) area-normalized dose for the cream (left) and ointment (right), and for each subject before and after counselling. Participants are grouped into those who had previously used topical steroids (*n* = 9) and those who had not (*n* = 15). The dotted lines in panel (**c**) indicate the normalized doses used for the in vivo tape stripping and the in vitro permeation studies. Asterisks (*) indicate significant effects of counselling on the dose expressed (*p* < 0.001) and area of spread (*p* < 0.005). Paired superscript letters identify values significantly different for the cream and the ointment.

Because neither of the PILs explained FTUs and some subjects did not read them (Table 1), groups A and B behaved in the same way, so the data were pooled for analysis. Three Two-Way ANOVAs (Prism 5, GraphPad Software Inc., La Jolla, CA, USA, 2017) explored the effects of “counselling” and “previous TCS use” on the “length of dose measured”, the “area of spread” and the “ND” applied. Paired *t*-tests explored whether the “area of spread” and the “ND” were (a) modified after counselling for a given group and formulation and (b) differed for the cream and ointment in a given group (Figure 2). The criteria for statistical significance were fixed at *p* < 0.05.

### 2.3. In Vivo Tape-Stripping Experiments

The study was approved by the ethics committee for the School for Health of the University of Bath (REACH, EP13/14107). Six healthy subjects (Table 1) provided written informed consent before participation. The subjects had neither history of dermatological disease nor recent topical formulation use, and refrained from physical activity and bathing of the arms for the duration of the experiment. The tape-strips from the first participant were only used for analytical development. The forearms were washed briefly (~5–10 s) with tepid water, dried gently with cellulose paper and allowed to dry for 15 min. This step aimed to ensure that the starting skin condition was clean and the same for all subjects.

Eight skin sites (4.9 cm^2^), four on each forearm, were demarcated with adhesive foam (3 M, Minnesota, MN, USA). The 4 sites on each forearm were positioned at least 5 cm from the wrist and 5 cm from the elbow. One forearm was used for uptake samples and the other for clearance samples. Each formulation was applied once thinly (2 mg/cm^2^) and once thickly (9 mg/cm^2^) on each forearm. Application of each combination (formulation/ area-normalized dose) was staggered by 30 min. The location of the four combinations (formulation/area-normalized dose) was paired for the forearms of each participant but rotated among subjects to account for skin variability along the forearm. The formulations were applied using a tared inverted vial and the exact dose applied calculated by weight difference. The sites were covered with non-occlusive, sterile gauze. All formulations were removed 6 h after application using three Sterets^®^ (70% isopropyl alcohol) wipes (Molnlycke Health Care, Lancashire, UK) as described in previous work [15,16]. Uptake sites were left to dry for at least 5 min before tape-stripping. The clearance sites were protected with non-occlusive gauze [15] and tape-stripped 24 hs after application. This 18-h clearance was chosen so the HC loss from the SC would reflect primarily drug absorption deeper into the body rather than loss by desquamation.

Skin sites were delimited by a circular template (3.14 cm^2^), their baseline transepidermal water loss (TEWL_baseline_) measured (Biox Aquaflux AF200, BiOX, London, UK) and tape-stripped using adhesive tapes (7.5 cm^2^, Scotch Book Tape 845, 3 M). Tape-stripping continued until the first of four stop criteria was met: 6xTEWL_baseline_, TEWL ≥ 60 g m^2^ h^−1^, 30 tapes or evidence of SC ‘clumping’ (localized clump of many layers of SC removed on a single tape). Additionally, the depth of the SC was measured at one untreated site using the baseline-corrected non-linear model [17] in which case TEWL was measured after each tape removal.

The SC depth reached with each tape was determined from the area, mass gain on tapes and density of 1 g cm^−3^ [18]. The tapes were discharged from static electricity (R50 discharging bar and ES50 power supply, Eltex Elektrostatik GmbH, Weil am Rhein, Germany) and weighed on a microbalance (Sartorius SE-2F, Sartorius AG, Göttingen, Germany; precision 0.1 µg). The mass was taken as zero when the weight after tape-stripping was less than before tape-stripping.

Analytical: Tapes were rolled into a vial with 1 mL of 60:40 methanol:water mixture and sonicated for 60 min at 80% power at room temperature. The efficiency of the extraction method [19] was determined as 97.9% in our experimental conditions. With exception of the first two, the tapes were extracted in groups of three or four (combined SC mass ≥0.4 mg) to improve analytical sensitivity [15]. HC was quantified by HPLC (Shimadzu LC-2010AHT with LCMS solutions software, Shimadzu, Kyoto, Japan) with UV detection (245 nm). A 55:45 methanol:water mobile phase was pumped (1 mL min^−1^) through a 25 cm C18 column with a C18 4 mm × 2 mm security guard cartridge (Phenomenex). The injection volume, retention time and limit of quantification were 75 µL, 15 min and 0.1 µg mL^−1^, respectively.

**Figure 3:**
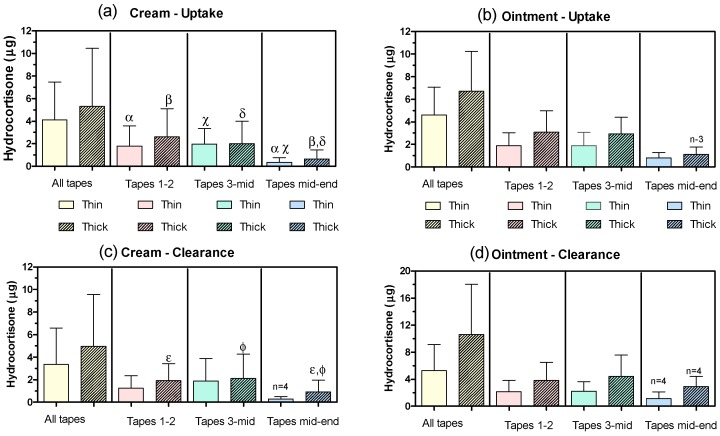
Mean (±SD, *n* = 5 except indicated otherwise) hydrocortisone (HC) recovery for (**a**) uptake-cream; (**b**) uptake-ointment; (**c**) clearance-cream and (**d**) clearance-ointment samples following thin (empty bars) and thick (hatched bar) application. All tapes (yellow bars); T_1–2_ (pink bars); T_3-mid_ (green bars) and T_mid-end_ (blue bars). The results from eight ANOVAs used to assess differences in HC across three sections of the stratum corneum (SC) are shown; the pairs of values which are significantly different (*p* < 0.02) are identified by the matching Greek symbols (α, β, χ, δ, ε and ϕ). Note the different Y-axis range used for the ointment-clearance data set.

Data analysis: The depth sampled was normalized by the SC thickness measured at the untreated site. The amounts of HC recovered from all tapes, the first two tapes (T_1–2_), the third to mid-depth tape (T_3-mid_) and the mid-depth to last tape (T_mid-end_) were calculated. The “mid-depth” was allocated to the group including the deepest tape reaching a normalized depth <0.5. First of all, eight One-Way ANOVAs compared the content of HC in SC sections (All tapes, T_1–2_, T_3-mid_, and T_mid-end_) for each treatment (product and normalized dose (ND)) and sampling time (uptake/clearance) (Figure 3). Next, a series of Two-Way ANOVAs explored whether HC content in SC sections (All tapes, T_1–2_, T_3-mid_ and T_mid-end_) was modified by (a) the “sampling time” and “ND” for the cream and the ointment and, (b) by the “formulation” and the “ND” at uptake and clearance times. Whenever possible, paired Two-Way ANOVAs were used which were followed by Newman–Keuls and Bonferroni post-tests as appropriate.

Finally, the [thick/thin]ratios or (HC recovered from SC with thick application)/(HC recovered from SC with thin application) were calculated and compared to a reference value of 1 using the “one sample *t*-test” provided by Prism 5 (GraphPad Software Inc.).

### 2.4. In Vitro Permeation Tests (IVPT)

IVPT using Franz diffusion cells (3.14 cm^2^, 37 °C, *n* = 6) investigated the effect of thin (2 mg/cm^2^) and thick (9 mg/cm^2^) dosing on the permeation of HC across dermatomed porcine skin into a PBS receptor over 26 h. The formulations were applied using a tared inverted vial and the exact dose calculated by weight difference. Receptor samples (1 mL) were taken at various time points (2–26 h), filtered and HC quantified by HPLC (as above) with a 60:40 methanol:water mobile phase. Neither the SC nor the viable skin were analyzed for HC content at the end of these experiments.

## 3. Results

### 3.1. Inter-Subject Variability in Dosing Topical Products

Table 1 summarizes the key features of the participants in the study. The questionnaire found that subjects’ specialism (i.e., profession, degree undertaken) did not provide knowledge of FTU so no participant was excluded. Nine of the participants in the dosing study had used TCS previously and their results were analyzed separately (“previous use”). For both formulations, the dose measured and the area of application differed between naive and “previous use” participants, and changed after the counselling intervention (Figure 1 and Figure 2). Previous TCS users measured longer doses (*p* < 0.01) and spread them over larger areas (*p* < 0.02). Both the area of application (*p* < 0.02) and the dose measured (*p* < 0.0001) increased after counselling. Normalized doses (NDs) decreased after the intervention (Figure 2) but the effect was not significant. Finally, NDs were larger (*p* < 0.01) for the ointment than for the cream both before and after counselling.

### 3.2. In Vivo Tape-Stripping Experiments

Table 2 and Figure 3 summarize the tape-stripping results. All the participants (*n* = 5) completed the study although fewer replicates were available for the T_mid-end_ section at some skin sites because the criteria to stop tape-stripping had been reached. Given the consistent dose applied and SC depth sampled (Table 2), it was possible to compare formulations, and sampling (uptake and clearance) times.

A first series of One-Way ANOVAs analyzed the distribution of HC across the SC (Figure 3). The content of HC in the deepest section (T_mid-end_) of the uptake samples was significantly less (*p* < 0.02) than in more superficial layers (T_1–2_, T_3-mid_) following thin and thick applications of the cream. In the case of clearance samples, the same pattern was observed only for thick cream applications (Figure 3). When the ointment was used, no significant differences were found among the HC content in the three SC sections considered.

Next, two series (one series per sampling time) of Two-Way ANOVAs compared whether the HC recovered from different SC sections was modified by the factors “formulation” and “normalized dose”. Overall, and independently of the formulation used, thicker applications led to significantly more HC in uptake samples when the whole SC (all tapes, *p* < 0.04) and T_1–2_ (*p* < 0.02) were considered. In the case of clearance samples, the effect was only significant for the deepest tapes (T_mid-end_) (*p* < 0.03). No formulation effects were significant for uptake samples whereas the T_mid-end_ tapes at clearance time had more HC (*p* < 0.02) when the ointment was applied.

Finally, another two series (one series per product) of Two-Way ANOVAs compared whether the HC recovered from different SC sections was modified by the factors “sampling time” and “normalized dose”. Thicker applications of the cream led to higher (*p* < 0.02) recovery from the first two tapes only. In the case of the ointment, thicker applications resulted in higher recoveries from T_3-mid_ and T_mid-end_ that narrowly missed (*p* = 0.06) the significance threshold (*p* ≤ 0.05). The differences between HC content in the SC at uptake and clearance times did not reach statistical significance in any case.

The [thick/thin] ratios were 1.6 ± 1.0 (cream-uptake); 1.4 ± 0.5 (ointment-uptake); 1.9 ± 0.9 (cream-clearance) and 2.5 ± 2.1 (ointment-clearance). These values were not statistically significant different from a hypothetical value of one.

### 3.3. In Vitro Permeation Experiments

Table 3 summarizes the IVPT results. After 26 h, HC was detected in the receptor of only one (cream) and of none (ointment) of the six replicates for the lower ND (2 mg/cm^2^). Thicker applications (9 mg/cm^2^) resulted in greater cumulative delivery at 26 h for the cream (*p* < 0.01) and measurable permeation for the ointment. In the case of thicker applications, there were not significant differences between the two formulations.

## 4. Discussion

This work investigated whether variability in the area-normalized dose of TCS has a significant effect on the skin absorption of HC. A first step established the dosing behavior, approach and variability, in subjects prior to, and after, a short counselling on the use of FTUs. In addition, the study assessed whether patients applied more consistent doses of TCS after being advised on FTUs. Whilst under-dosing and poor adherence to TCS are well documented [5,7,8], little is known about the effectiveness of dosing approaches such as FTUs in improving TCS dosing, reducing variability, and any potential effects on HC absorption.

Correct dosing with FTUs involves measuring the right length of formulation and spreading it sufficiently on the skin. Before counselling, most participants underestimated the amount of product corresponding to one FTU (Figure 2). Indeed, some participants put some formulation literally on the “tip of their fingers”. Participants with TCS experience measured a dose corresponding to 63% (cream) and 55% (ointment) of their average fingertip length. Underdosing was more noticeable for participants naïve to TCS who measured 36% (cream) and 35% (ointment) of the length required. This value is consistent with prior estimations that dermatologic patients apply ~35% of the expected dosages [7]. After the short (<1 min) counselling, the participants measured longer doses with less variability. Indeed, the length expressed from the tube was comparable (99–114%) to the average fingertip (Figure 2; Table 1). Thus, a first finding of this study was that a very short intervention explaining FTUs could alleviate underdosing and would be beneficial for patients and carers, including those having used TCS previously.

The second dosing step required the subjects to spread the formulations sufficiently, so the ND approached the 1.5 mg/cm^2^ goal [10]. However, this target does not take into account the potential weight loss by evaporation of excipients occurring upon application. We determined that about 15% of the weight of cream applied was lost in 15 min so correct application of one FTU would yield a value closer to 1.3 mg/cm^2^. Before counselling, and despite the insufficient doses measured, the participants did not spread the products enough so the NDs exceeded the target (Figure 2). Afterwards, the participants measured larger doses and spread them further so there was a net, albeit not significant, decrease in ND. It was observed that smaller (*p* < 0.02) areas were used for the ointment, maybe reflecting the cream’s better spreadability. Consequently, with the one exception (previous use before counselling), NDs were greater (*p* < 0.02) for the ointment.

Overall, counselling reduced ND from 4.7 ± 8.7 mg/cm^2^ to 2.0 ± 2.4 mg/cm^2^. Further, before counselling, four (cream) and 13 (ointment) participants applied more than 3 mg/cm^2^; reduced to two (cream) and seven (ointment) participants post-intervention. Thus, whilst most subjects applied relatively acceptable ND, the short counselling would particularly support users with poor dosing technique. Indeed, the intervention reduced the maximum ND from 37 mg/cm^2^ (cream) and 48 mg/cm^2^ (ointment) to 8 mg/cm^2^ (cream) and 14 mg/cm^2^ (ointment).

These values provide an estimation of the average and range of NDs applied by patients with and without counselling, information currently not available. However, they should be cautiously extrapolated to clinical scenarios where the presence of skin lesions might modify the dosing approach. Even so, this study estimated the variability associated with dosing TCS so its potential impact on HC absorption was investigated next. The 4.5-fold (2 mg/cm^2^ to 9 mg/cm^2^) range in ND chosen for the in vivo study provided a reasonable expectation for effects without increasing the volunteers’ exposure unnecessarily. In addition, the range comprised all the average NDs and all but six of the 96 individual NDs measured in the first study (Figure 2), thus it would encompass the NDs applied by most patients.

The vasoconstrictor assay was not used because of the poor skin blanching effect of HC in the study conditions [20,21,22,23,24]. Instead, tape-stripping, a technique proposed as a dermato-pharmacokinetic approach that has provided results consistent with the vasoconstriction assay, was chosen as a surrogate test for skin absorption [15,21,22,25,26]. In addition, tape-stripping has advantages over the vasoconstrictor assay such as exhibiting a linear response, not requiring selection of responders and, as demonstrated by this study, allowing discrimination between formulations, doses and exposure times that cannot be tested using the blanching test.

**Figure 4:**
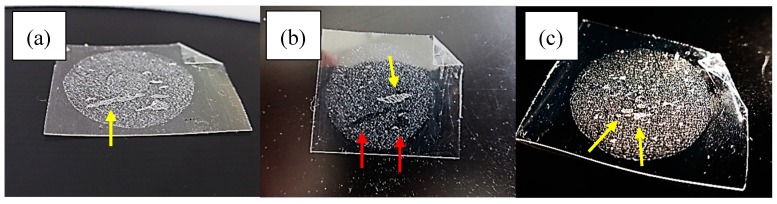
Inhomogeneous tape-stripping observed for subject 5 (**a**) and (**b**) and subject 4 (**c**). Panel (**a**) the yellow arrows indicate areas where clumps of corneocytes were removed by the tape. Panel (**b**) A subsequent tape removed clumps of corneocytes in other sites (yellow arrows) but collected little SC in darker regions (red arrows) that matched the sites where clumping was observed in preceding tapes. Panel (**c**) Example of the inhomogeneous tape stripping observed in another volunteer.

The average HC recovery from the SC, ~5 µg (1.6 µg/cm^2^) was consistent with previous reports (1.5–3.5 µg/cm^2^) [20]; the contribution of endogenous HC to this value is probably negligible [27]. Comparison with prior experiments not involving formulation removal before tape-stripping [21] suggested that the cleaning method had efficiently removed the formulation from the skin surface [16]. Our cleaning approach was based on Wiedersberg et al. [16]) who showed that IPA (isopropanol alcohol ) cleaning led to lower amounts of drug (betamethasone valerate) recovered from the tapes as compared to cleaning with cellulose paper, therefore suggesting that IPA wipes were a more efficient cleaning approach. However, the diffusion parameter (D/L^2^) estimated for the drug was independent of the cleaning method, suggesting that IPA cleaning had not “pushed” drug into the SC. A greater HC content was expected for the first two tapes (T_1–2_) which sample more SC from the superficial layers that contain higher drug concentration [28,29] although the differences were only significant for the cream (Figure 3). The data in Figure 3 do not represent exact “HC concentration versus SC depth” profiles for several reasons: first of all, and due to the skin furrows, the adhesive tapes may sample cell layers originating from different SC depths [30]; second, because the tapes were grouped to improve analytical sensitivity [15] and finally, due to formulation effects on the corneocytes cohesion [30]. Indeed, the uptake tapes from four out of six subjects showed clear “clumps” or noticeable thicker SC regions in the tape, assumed to correspond to multiple layers of corneocytes (Figure 4). The evidence of clumping was used as criteria to stop tape-stripping to limit the invasiveness of the procedure. A final finding consistent with the so-called “hydrocortisone reservoir effect” [19,31] was that the amounts of HC in uptake and clearance samples (Table 2) were not significantly different. It has been proposed [26] that the difference in the amounts of drug recovered from the SC at uptake and clearance sampling times reflects the amount of drug ultimately absorbed into the viable tissue and potentially, systemically. The results of the study suggest that little HC is absorbed systemically after application of one dose over the range of NDs tested.

Overall, more HC was recovered from sites treated with higher ND (All tapes-uptake, tapes 1–2—uptake, and tapes mid-end—clearance). There were no formulation effects, as expected for products considered interchangeable. Separate analysis for each formulation suggested that thicker applications of the cream primarily modified the amount of HC in the first two tapes whereas thicker applications of the ointment led to higher HC recoveries from the T_3-mid_ and T_mid-end_.

The [thick/thin]ratios (see Section 3) were typically higher than, but not significantly different to 1. Therefore, they did not reflect the 4.5-fold increase in the normalized dose applied. The dosing variability in the range studied had relatively minor effects on the SC permeation of HC and potentially, its systemic absorption. Thus, patients could be reassured by healthcare professionals accordingly. Nevertheless, the role of other factors such as total area of skin treated, impact of multiple doses, age of patient, status of the skin barrier function, and potency of the specific corticosteroid should also be considered to provide individualized advice.

The significant, albeit modest, increase in the 6-h uptake of HC observed for thicker applications is consistent with previous work [12,13,14] predicting that thinner applications deplete more rapidly, leading to declining fluxes whereas thicker dosing will deliver the active for longer periods. Indeed, the percentage of HC dose applied recovered from the SC was higher for thinner (6.5% cream, 7.3% ointment) than thicker (1.9% cream, 2.4% ointment) applications. This prior work [12,13,14] also predicts that longer exposure times would discriminate better between the two normalized doses. Thus, a 26-h long in vitro permeation test was performed using dermatomed porcine skin, a model proven useful in studies that compared the permeation fluxes, partitioning and diffusion parameters of ibuprofen across human and porcine skin [28]. The in vitro permeation data corroborates this hypothesis (Table 3). After 6 h, HC was found in the receptor only when thicker applications of the cream were used. As seen in recent work [26], the in vivo tape-stripping and the in vitro permeation results at 6 h led to the same conclusions overall. However, after 26 h, the effect of the formulation thickness was clearer and ~7 times more HC was found in the receptor when the larger ND was used. The differences were more manifest for the ointment as no HC was detected in the receptor following thin applications.

To summarize, thicker applications resulted in larger SC-uptake of hydrocortisone in vivo and cumulative delivery in vitro. The differences observed after a 6 h exposure were relatively minor and did not reflect the 4.5-fold increase in normalized dose. Longer exposures led to larger differences in agreement with predictions. The results of this study support that despite dosing variability, systemic risks associated with HC treatment are low. Further evidence should be gathered from a multiple dosing in vivo tape-stripping study that mimics chronic use of TCS.

## 5. Conclusions

Brief counselling in correct application of fingertip units of topical products was an effective tool to minimize variability and limit under-dosing of TCS, and was useful for subjects with and without previous TCS experience. A tape-stripping in vivo study showed that thicker (9 mg/cm^2^) applications of a 1% hydrocortisone cream and of an ointment increased the drug uptake by the stratum corneum compared to thinner (2 mg/cm^2^) dosing. These observations were further supported by in vitro studies over longer permeation times. However, following a 6-h exposure, the differences were relatively small and not proportional to the increase in the normalized dose applied. The results of this study provide additional support that systemic risks associated with HC treatment are low even when dosing variability by patients and carers is considered.

## Figures and Tables

**Table 1:** Key descriptors of the two subject populations that participated in the two studies.

Descriptor	FTU Dosing Study	Tape-Stripping Study
Age (years)	27.2 ± 10.3	26.0 ± 3.7
Gender	14 male; 10 female	3 female; 2 male
Fingertip length (mm)	28 ± 3	Not applicable
Previous use of TCS	9 yes; 15 no	No recent use
PIL offered?	12 yes; 12 no	Not applicable
If yes; PIL read?	8 yes; 4 no	

FTU: fingertip unit; TCS: topical corticosteroid; patient information leaflets.

**Table 2:** Average (±SD, *n* = 5) normalized doses (ND) applied of cream and ointment; HC (hydrocortisone) recovery from SC (stratum corneum) and SC depth sampled for uptake and clearance samples. The area of skin tape-stripped was 3.14 cm^2^.

Formulation	Uptake			Clearance		
	ND (mg/cm^2^)	HC in SC (µg)	SC Depth (µm)	ND (mg/cm^2^)	HC in SC (µg)	SC Depth (µm)
**Cream**	2.3 ± 0.2	4.11 ± 3.36	11.1 ± 3.6	2.3 ± 0.2	3.36 ± 3.23	9.9 ± 4.4
	9.2 ± 0.5	5.30 ± 5.17	11.4 ± 2.4	9.3 ± 0.7	4.97 ± 4.60	10.1 ± 2.4
**Ointment**	2.1 ± 0.1	4.60 ± 2.45	10.8 ± 3.7	2.2 ± 0.1	5.27 ± 3.87	11.5 ± 4.0
	8.9 ± 0.4	6.70 ± 3.53	8.0 ± 1.9	9.4 ± 0.2	10.6 ± 7.43	11.7 ± 4.5

**Table 3:** Mean (±SD, *n* = 6) in vitro cumulative HC delivery across dermatomed porcine skin from thin (2 mg/cm^2^) and thick (9 mg/cm^2^) area-normalized doses of 1% HC cream and ointment. The transport area was 3.14 cm^2^. The superscript ^γ^ indicates significantly different values (*p* < 0.01).

Formulation	Normalized Dose (mg/cm^2^)	Cumulative HC in Receptor (µg)			
		6 h	22 h	24 h	26 h
**Cream**	2.3 ± 0.2	Not detected	0.19 ± 045	0.15 ± 0.38	0.17 ± 0.40 ^γ^
	9.8 ± 1.2	0.16 ± 0.19	1.22 ± 1.42	1.27 ± 1.47	1.31 ± 1.52 ^γ^
**Ointment**	2.4 ± 0.4	Not detected	Not detected	Not detected	Not detected
	9.4 ± 0.5	ND	2.22 ± 3.17	2.27 ± 3.22	2.41 ± 3.38
